# Can Vitamin D Deficiency Increase the Susceptibility to COVID-19?

**DOI:** 10.3389/fphys.2021.630956

**Published:** 2021-05-21

**Authors:** Quratulain Maha, Muhammad Talal

**Affiliations:** ^1^Medical College, The Aga Khan University, Karachi, Pakistan; ^2^Medical College, Dow University of Health Sciences, Karachi, Pakistan

**Keywords:** vitamin D deficiency, immunity, cytokine storm, vitamin D, COVID-19

## Introduction

**Co**rona **vi**rus **d**isease of 20**19** (COVID-19) is the infectious disease caused by the novel coronavirus SARSCoV-2 (severe acute respiratory syndrome coronavirus 2) (Lai et al., 2020). The first case was reported in Wuhan, China, in the December of 2019 (Lai et al., 2020). In March 2020, the WHO declared COVID-19 as a pandemic and to this date (i.e., 6th April 2021) 131,487,572 cases and 2,857,702 deaths have been recorded by the WHO worldwide (WHO Coronavirus Disease Dashboard, 2020). Therefore, it is necessary to educate the public regarding preventive measures, and healthcare professionals regarding accurate and up to date knowledge that help them manage and treat patients. Here we talk about the possible role of Vitamin D in COVID-19 based on current scientific evidence.

## Pathogenesis of COVID-19

Coronaviruses are a large family of viruses. The first known severe illness caused by a coronavirus was Severe acute respiratory syndrome (SARS)-CoV, (started in China in 2002), and the second one is the ongoing Middle East respiratory syndrome (MERS)-CoV (started in the Middle East in 2012; Grant et al., 2020). These are amongst the previous CoV epidemics.

The disease spans from; a symptomless stage (which includes almost 50% of the patients) where the patient shows no symptoms, to, a critical stage (5% of the symptomatic patients) that involves high mortality rates due to severe involvement of the lungs along with diffuse damage to alveolar tissue and destruction of pulmonary tissue (Wang et al., 2020) that leads to Acute Respiratory Distress syndrome (ARDS) (Tian and Rong, 2020a). The critical stage also includes multi- organ dysfunction and/or shock (Wang et al., 2020). Whether a patient progresses from the asymptomatic stage to other stages (mild, moderate, and severe) depends on many factors some of which include diabetes, hypertension, pre-existing heart disease, old age, male sex, obesity, the immune status of the person, and COVID-19 associated coagulopathy (CAC) (Lau et al., 2020; Wang et al., 2020).

SARS-CoV-2 binds on to the Angiotensin Converting Enzyme 2 (ACE2) to enter in to human cells (Zhang et al., 2020). This metallopeptidase is expressed not only in the respiratory epithelium but also in the endothelium of vessels, intestines, heart, and kidney (Zhang et al., 2020). The SARS-CoV spike (S) protein has subunits; the **S1** subunit is responsible for receptor binding while the **S2** subunit is responsible for fusion of the viral envelope with the host cell membrane (this is how it facilitates viral entry in the cell) (Heurich et al., 2014). This S protein has a strong affinity for the catalytic domain of ACE2 (Heurich et al., 2014; Zhang et al., 2020). The binding of ACE2 to the S protein causes certain conformational changes which result in the cleavage of the S protein; a cleavage that is essential for viral infectivity (Heurich et al., 2014). The type II transmembrane serine proteases (TMPRSS2) and human airway trypsin-like protease (HAT), can cleave and activate the S protein as well (Heurich et al., 2014). Both TMPRSS2 and HAT are expressed in ACE2-positive cells in the human lung. And it is also suggested that TMPRSS2 might play a significant role in SARS-CoV spread in the human respiratory tract (Heurich et al., 2014).

A more recent study suggested that ACE2 is also processed by TMPRSS2 and HAT, and it was suggested that ACE2 cleavage increases SARS-CoV-2 S-protein mediated entry into the host cells (Heurich et al., 2014). It was identified that the amino acid residues from 697 to 716 (of arginine and lysine) were essential for this cleavage by TMPRSS2 and HAT (Heurich et al., 2014). Further analysis and evaluation suggests that SARS-CoV-2 recognizes the human ACE2 much more efficiently than SARS-CoV, which increases the transmissibility of SARS-CoV-2 from one person to another (Zhang et al., 2020).

This helps us understand how the virus may attack and damage blood vessels, which eventually leads to the formation of thrombi (Tian and Rong, 2020b) and, this might be linked to mortality (Zhang et al., 2020). This is the reason why vascular injuries have now become a center of attention in COVID-19. Besides the virus virulence, COVID-19 is caused by the release of pro-inflammatory cytokines, in particular Interleukin 6 (Khan et al., 2021). These cytokines cause recruitment of monocytes/macrophages and neutrophils (Khan et al., 2021). These cells are capable of destroying invaders directly by phagocytosis and increase production of pro-inflammatory cytokines causing a cytokine storm or cytokine release syndrome (CRS), which is also known as Macrophage Activation Syndrome (MAS), that can cause increased morbidity and mortality (Grant et al., 2020; Khan et al., 2021).

## Vitamin D and Immunity

Vitamin D, being one of the four lipid soluble hormones, is considered to be a pro-hormone. While a small percentage is acquired from diet such as oily fish, red meat, and egg yolk (Vitamin D2), most of it is produced in the skin (Vitamin D3) when Ultraviolet radiation (UVB radiation at 290–315 nm) strikes and 7-Dehydrocholesterol (7-DHC) which is a cholesterol derivative, is converted to pre-vitamin D3, which is then non-enzymatically isomerized to Vitamin D. It is then hydroxylated in the liver by 25-hydroxylase (CYP2R1), to form 25-hydroxyvitamin D (25-OHD) which is the main circulating form of vitamin D. This circulating metabolite of vitamin D tells about the vitamin D status of a person. This is then hydroxylated by 1α-hydroxylase (CYP27B1) in the kidney to 1, 25 di-hydroxyvitamin D (1, 25-OHD), the metabolically active form of vitamin D (Gois et al., 2018; Maha et al., 2019; Lanham-New et al., 2020). Though there is no universally agreed definition, Vitamin D deficiency (VDD) in UK is taken as a 25-OHD concentration of <10 ng/ml (25 nmol/L), whereas in the US it is taken as <12 ng/ml (30 nmol/L). Vitamin D insufficiency (VDI) is mostly considered between 25 and 50 nmol/L, and sufficiency is >50 nmol/L of 25-OHD (Thacher and Clarke, 2011).

Where vitamin D is usually thought of improving bone health, it can do much more. It can reduce the risk of infections by playing its role in improving immune function. The expression of the nuclear vitamin D receptor (VDR) and presence of vitamin D metabolic enzymes such as 1α-hydroxylase (CYP27B1) in the cells of immunity such as macrophages, and the identification of numerous primary 1, 25-OHD target genes in immune cells substantiates its role in maintaining immune homoeostasis (Chun et al., 2014). The inflammatory cells up-regulate VDR and promote the conversion of vitamin D metabolites to 1, 25-OHD (Maha et al., 2019).

According to studies, monocytes and macrophages have been identified as one of the non-renal cells with the potential to synthesize 1, 25-OHD, and also up-regulate 1α-hydroxylase expression. The intracellular system for vitamin D activation in monocytes comprises of the conversion of 25-OHD to 1, 25-OHD by the mitochondrial 1-α-hydroxylase. This then acts as a transcription factor for antimicrobial peptides such as cathelicidins (CAMP) and defensins such as β-defensin 4A (DEFB4) by binding to the cytoplasmic VDR (Gois et al., 2018; Wichmann et al., 2020). The machinery for vitamin D activation was also recently observed in other antigen-presenting cells (APCs) such as Dendritic Cells (DC) (Gois et al., 2018).

A study revealed that some patients with Sarcoidosis (a granulomatous disease) were shown to have increased levels of 1, 25-OHD due to conversion of 25-OHD to 1, 25-OHD by 1α-hydroxylase in tissue and systemic macrophages (Chun et al., 2014). Such observations have been made for other inflammatory and granulomatous diseases such as Tuberculosis. Pattern Recognition Receptors (PRR) such as Toll- Like receptors (TLR) sense Mycobacterium tuberculosis and signal to induce expression of 1α-hydroxylase and VDR in monocytes and macrophages (Chun et al., 2014). However, some *in vitro* studies revealed the potential for macrophage 1α-hydroxylase activity in the absence of disease, thus suggesting that vitamin D activation is part of a normally functioning immune system (Chun et al., 2014).

Vitamin D's role in immunity can be put into three categories: physical barrier, innate immunity, and adaptive immunity. It helps maintain tight junctions, gap junctions, and adherens junctions (e.g., by E-cadherin) (Grant et al., 2020) among the epithelial cells thereby enhancing the physical barrier which is the first barrier encountered by any pathogen (bacterial or viral etc.). Our immune system has two arms: Innate and Adaptive. Vitamin D plays a potent immune-modulatory role in the cells of both innate as well as adaptive immunity (Chun et al., 2014; Wichmann et al., 2020). This way it enhances innate defense mechanisms against pathogens.

Vitamin D also affects several TLRs (Ilie et al., 2020). TLRs play a crucial role in the innate immunity. Pathogens display certain molecules (pathogen-associated molecular patterns-PAMPs) that are recognized by the PRRs (such as TLR). When these receptors are activated, they release cytokines and induce reactive oxygen species (ROS) and antimicrobial peptides (as mentioned above; Chun et al., 2014; Grant et al., 2020). These peptides, such as cathelicidin, work against the pathogens by disturbing their cell membranes and neutralizing endotoxins etc. and hence help reduce the viral load (and its virulence). Their antimicrobial spectrum includes Gram-positive and Gram-negative bacteria, enveloped and non-enveloped viruses, and fungi (Grant et al., 2020). Cathelicidins are known to have anti-bacterial properties through its *in-vitro* suppression of Mycobacterium tuberculosis (Ilie et al., 2020; Tian and Rong, 2020b). A study on critically ill patients with severe sepsis found significantly lower concentration of vitamin D associated with low antimicrobial peptide cathelicidin (Jakovac, 2020).

Innate immunity includes the production of both pro-inflammatory and anti-inflammatory cytokines. Vitamin D modulates a set of white blood cells [type 1 T-Helper (Th1) cells] by preventing them from releasing too many inflammatory cytokines namely Tumor Necrosis Factor α (TNF-α), Interferon- γ (INF-γ), and Interleukin-6 (IL-6). These pro-inflammatory cytokines play a part in the recruitment of neutrophils and macrophages etc. that cause inflammation and injure the lungs. Vitamin D has immunosuppressive properties as it reduces the function of innate immunity by reducing cytokine mediated responses particularly down-regulation of IL-12 and up- regulation of IL-10-mediated responses (Panarese and Shahini, 2020). It also modulates adaptive immunity by promoting type 2 T helper (Th2) cells to produce cytokines which indirectly suppress Th1 cells. Vitamin D also promotes the induction of T regulatory cells which help to inhibit inflammatory processes (Grant et al., 2020; Tian and Rong, 2020a).

Vitamin D also plays a role in the regulation of innate immune responses mediated by macrophages and DCs, which are the first line of host defense. It prevents the macrophages from releasing too many inflammatory cytokines and chemokines, and increases the expression, and thus, production of anti-inflammatory cytokines (Tian and Rong, 2020a). It is also noted that Vitamin D impacts the maturation of T cells such that it prevents inflammatory Th-17 phenotype. Moreover, it inhibits the maturation and differentiation of DCs while preserving immature phenotypes as witnessed by a decreased expression of MHC class II proteins, co-stimulatory molecules and IL-12 (Aranow, 2011). Supplementation of Vitamin D has proven to augment the process of phagocytosis of monocytes and induce macrophage autophagy (Konijeti et al., 2016). This is how it reduces the cytokine storm, which is seen in COVID-19 infected patients. Therefore antiviral effects of vitamin D include direct interference with viral replication, and acting as an immune-modulatory and anti-inflammatory agent (Teymoori-Rad et al., 2019).

Moreover, some studies have also reported the expression of VDR, 1α-hydroxylase and 24-hydroxylase in human B Lymphocytes. Some studies also indicate that 1, 25-DOH may impede the differentiation of B cells to plasma cells, thus modulating the production of antibodies (Gois et al., 2018).

## Vitamin D and COVID-19

With many researches proving vitamin D's role in preventing acute respiratory tract infections (ARTIs) (Martineau et al., 2017; Ilie et al., 2020) and other viral infections (Jiménez-Sousa et al., 2018; Martínez-Moreno et al., 2020), it might help fight off COVID-19 too. Vitamin D has been shown to decrease the risk of respiratory tract infections and/or severity of respiratory infections (Martineau et al., 2017). It increases the levels of ACE-2 which is known to have a protective role against acute lung injury (Ilie et al., 2020). It has also been shown to attenuate lipopolysaccharide-induced acute lung injury in mice by modulating effects on the renin-angiotensin system (RAS) and ACE2/Angiotensin signaling pathway (Panarese and Shahini, 2020). Higher levels of ACE2 have also been associated with better COVID health outcomes in previous studies (Ilie et al., 2020).

As we mentioned above, ACE2 is known to be a target of SARS-CoV-2, mediating viral entry into target cells. In a study, overexpression of human ACE2 in mice with SARS-CoV infection increased the disease severity proving that the entry of virus in cells is an important step in the pathogenesis of the disease (Zhang et al., 2020). Also, mice that were injected with SARS-CoV S-protein had an exacerbation in lung injury (Zhang et al., 2020). ACE2 helped attenuate this injury by blocking the renin-angiotensin pathway (Zhang et al., 2020). Thus, ACE2 is not just the receptor involved in viral entry but it is also a protective agent for the lung.

As discussed earlier, vitamin D modulates the immune responses and prevents the release of pro-inflammatory cytokines. These are the same cytokines that eventually cause CRS (seen in critical COVID-19 patients).

In order to have proper vascular function, it is imperative to have adequate levels of Vitamin D metabolites (Lau et al., 2020). It promotes vascular endothelial repair by inducing the production of vascular endothelial growth factor (VEGF). It up-regulates the expression of thrombomodulin (TM), which is an anti-coagulant, and down-regulates the expression of tissue factor, which is an important coagulation factor (Lau et al., 2020). In a study on murine models, platelet aggregation was enhanced and gene expression of antithrombin (in the liver) and TM (in the aorta, liver, and kidneys) was down-regulated in VDR knockout mice, whereas, tissue factor expression in the liver and kidney was up-regulated in them (Aihara et al., 2004). Vitamin D also controls the expression (either directly or indirectly) of several genes responsible for a number of processes involved in the regulation of thrombosis-related pathways (Mohammad et al., 2019). Hence, VDD is prothrombotic and as we have seen, COVID associated Coagulopathy (CAC) has come to light as a key process in severe COVID-19. In a study in Wuhan, CAC was present in 71.4% of the non-survivors (Lau et al., 2020).

Vitamin D inhibits the renin-angiotensin-aldosterone system (RAAS) (Xu et al., 2017). Chronic activation of RAAS due to the vitamin's deficiency may lead to decreased lung function (Tian and Rong, 2020a). Keeping in mind the protective role of the vitamin through the regulation of the innate and adaptive immune response systems, as well as inhibition of RAAS, vitamin D supplementation might improve the immune system of COVID-19 patients and mitigate the disease in deficient individuals.

The fact that the outbreak of COVID-19 occurred during winters is highly suggestive of the role of Vitamin D in COVID-19 (winter is the time of lowest vitamin D, as measured by 25-OHD levels) (Kift et al., 2018); that there are less number of cases in the Southern Hemisphere (deaths and hospitalizations have occurred in 5.2 and 22% of patients in Northern latitudes, in 3.1 and 9.5% close to the Equator, in 0.7 and 8.7% in Southern latitudes, respectively) (Panarese and Shahini, 2020); that the mortality rate is currently higher in Northern latitudes, with Italy the highest (11.9%) (Panarese and Shahini, 2020) as in countries at high latitudes the UVB in winter isn't sufficient to produce vitamin D (Lanham-New et al., 2020). However a study showed that VDD is frequently under-diagnosed, mainly in countries where UV radiation is abundant such as Brazil (Unger et al., 2010), Iran (Maghbooli et al., 2020), etc. VDD has been found to contribute to ARDS as discussed above and that case-fatality rates increase with age and with chronic disease comorbidity, both of which are associated with lower vitamin D concentration (Grant et al., 2020). The group of population which is the most vulnerable for COVID-19, having the highest risk for morbidity and mortality with SARS-CoV2 (Lau et al., 2020); the elderly, also happen to be the one with low vitamin D levels (Ilie et al., 2020). This is because with increasing age vitamin D levels fall, mainly due to decreased exposure to sunlight and decreased cutaneous synthesis which is the main source, i.e., around 80–90% of the body's vitamin D comes from here (Ilie et al., 2020; Lau et al., 2020).

Other sources of the vitamin include fish and dairy products. These are found in high quantity in Scandinavian diets and this is why if we look at Europe, for example, the Scandinavian countries (Norway and Denmark) have VDI rates of 15–30% and COVID is much less severe here as opposed to Italy, Greece and Spain where VDI rates are around 70–90%, and the disease has been very severe here (Scharla, 1998). If we compare the serum vitamin D levels in the elderly with the prevalence of COVID, we can see that countries with high number of COVID-19 cases such as Italy and Switzerland have mean circulating Vitamin D levels of below 30 and 23 nmol/L respectively (Lau et al., 2020; Panarese and Shahini, 2020).

There have been studies that compared the prevalence of VDD in COVID-19 ICU patients. A study revealed that out of all the COVID-19 ICU patients in a setting, 84.6% of them were lacking adequate vitamin D levels (Lau et al., 2020). Another study compared the mean vitamin D levels of European countries and the number of COVID cases present there. It was found that countries that were most affected such as Italy, Spain had high rates of VDD. Whereas the Scandinavian countries were less affected as their diets include vitamin D rich foods (Panarese and Shahini, 2020) and they have a higher rate of vitamin D supplementation (van der Wielen et al., 1995). A recent study in Spain found that 80% of the 216 hospitalized COVID-19 were deficient in vitamin D (Hernández et al., 2020) and another one in Chicago concluded that people with VDD had 1.77 times higher risk of contracting COVID-19 (Meltzer et al., 2020). Recently a randomized trial in 76 hospitalized COVID-19 patients was done. The patients were randomized in a 2:1 ratio to vitamin D + best available COVID treatment at that time vs. this treatment without vitamin D. Of the 50 people in the vitamin D group, just one went to the ICU. Compared to 13/26 in the non-vitamin D group (Entrenas Castillo et al., 2020). These studies talk about VDD, but a recent one talks about less severity in COVID-19 symptoms in vitamin D sufficient patients. The study compared disease severity in two groups, vitamin D deficient and vitamin D sufficient, and saw that the later had reduced CRP levels, increased lymphocyte percentage, reduced clinical severity and hence reduced inpatient mortality (Maghbooli et al., 2020). One of the senior author of the study says, “This study provides direct evidence that vitamin D sufficiency can reduce the complications including the cytokine storm and ultimately death from COVID-19” (Maghbooli et al., 2020).

It is due to the above described mechanisms that supplementation with micronutrients like vitamin D, are thought to reduce risks of getting infection. Supplementation of Vitamin D greatly increases the expression of genes related to antioxidant production (e.g., glutathione reductase and glutamate-cysteine ligase modifier subunit). As glutathione production increases, it spares the consumption of Vitamin C from its role as an antioxidant, which has otherwise, antimicrobial activities. Amongst other micronutrients Vitamin C and Zinc supplementations are also proven to help boost immunity (Grant et al., 2020). Vitamin D supplementation has reportedly been of benefit in reducing severity of viral illness and inducing early recovery by several studies and maintenance of serum 25-OHD levels above 30 ng/mL (75 nmol/L) has been associated with good overall outcomes (Xu et al., 2017). But, the recommended dosage of vitamin D supplementation are not clear. Many guidelines recommend 600–4,000 IU/d and consider that a serum concentration of 20 ng/mL (50 nmol/L) is sufficient. But there have been arguments regarding the concentration, at some places it says that levels of 40–60 ng/mL (100–150 nmol/L) might benefit the highly predisposed groups for the viral infection and suggest taking 5,000 IU/d, after an initial 10,000 IU/d (which can sharply increase the concentration). Since the safety of high serum vitamin D levels is not certain, a serum level of 20–30 ng/mL (50–75 nmol/L) seems appropriate (Tian and Rong, 2020b), Vitamin D intoxication can be defined as 25-OHD levels of >150 ng/mL combined with other conditions like increased calcium levels in blood and urine (hypercalcemia and hypercalciuria, respectively) and increased phosphate levels in the blood (hyperphosphatemia). Therefore, in order to avoid the risk of intoxication levels above 125–150 nmol/L should be avoided (Gois et al., 2018).

In a meta-analysis it showed that regular oral vitamin D intake (in doses up to 2,000 IU/d without additional bolus), is not only safe but also has a protective role against acute respiratory tract infections, especially in people with VDD (Panarese and Shahini, 2020). People with Chronic Kidney Disease (CKD) are at a higher risk of developing VDD because the kidney loses its ability to convert 25-OHD to active 1, 25-OHD as the disease progresses further (Gois et al., 2018). For patients with stages 1-5 of Chronic Kidney Disease and Vitamin D deficiency or Vitamin D insufficiency, the supplementation guidelines are just the same as that for the general population. However, a variation is observed in different guidelines for optimal supplementation dosage. Some suggest 1,000–2,000 IU/d of vitamin D3 for vitamin D repletion, but they do acknowledge that CKD patients may require a more vehement therapeutic plan (Gois et al., 2018). A cross-sectional analysis of unselected patients with CKD stages 1-5, indicated that serum 25-OHD levels around 40–50 ng/ml may be a safe and efficacious treatment target in CKD, but additional studies are needed to examine this idea.

Another subject of controversy is about which form of vitamin D should be used. Vitamin D has two components: Vitamin D3 and D2 (Maha et al., 2019). The body uses both of them equally, using the same processes to convert them to the active 1, 25-OHD. A study comparing the potency of the two vitamin D analogs showed that both had similar initial increase in serum vitamin D levels, but people taking vitamin D3 had a better response. A meta-analysis concluded that vitamin D3 was more effective in incrementing serum 25-OHD levels, regardless of the dosage, frequency, or routes of administration (oral or intramuscular). According to some theories, vitamin D3 might have a higher affinity for both the receptor and enzyme (VDR and 25-hydroxylase), and even for vitamin D Binding Protein (VDBP) as compared to vitamin D2, thus leading to slower rates of clearance and eventually a longer half-life of vitamin D3 (Gois et al., 2018).

It is postulated that people with VDD are greatly predisposed to getting infected with COVID-19, or progress to severe stages of the disease when infected therefore vitamin D supplementation can possibly help to prevent and treat COVID-19. If, *per se*, Vitamin D does prove to have a role in prophylaxis or mitigation of the effects of COVID infection, supplementation with the vitamin could prove to be an easily affordable and minimal-risk intervention. However, so far no study suggests a promising role of vitamin D in the treatment of COVID-19. Though, a former director of the Center for Disease Control and Prevention (CDC), Dr. Tom Frieden, proposed using Vitamin D to combat the COVID-19 pandemic on 23 March 2020 (Former, 2020), further studies have to be done to prove its role in the treatment and management of COVID-19.

## Author Contributions

QM: writing—original draft, review, and editing. MT: writing—review and editing. Both authors contributed to the article and approved the submitted version.

## Conflict of Interest

The authors declare that the research was conducted in the absence of any commercial or financial relationships that could be construed as a potential conflict of interest.
