# Supplementary material for: Can Vitamin D Deficiency Increase the Susceptibility to COVID-19?
Source: Front Physiol. 2021 May 21;12:630956. doi: 10.3389/fphys.2021.630956 (PMC8176109; doi:10.3389/fphys.2021.630956)
Supplement: Supplementary file 1 [file Table_1.DOCX]

COVID -19 and Vitamin D table by Quratulain Maha and Muhammad Talal

| Vitamin D | References | COVID | References |
| --- | --- | --- | --- |
| Deficiency common in winters |  | Pandemic started in December | (1) |
| Insufficient UVB in northern latitudes in winter | (2) | High mortality rate in northern latitudes | (3) |
| Can reduce cytokine storm | (4) | Cytokine storm increases morbidity and mortality | (5) (6) |
| Reduces T helper cell inflammatory cytokine production | (7) | T helper cells release pro inflammatory cytokines | (7) |
| Reduces level of IL-6 | (7) | Proinflammatory cytokines ( IL-6, IL-12) cause recruitment of macrophages, neutrophils which lead to CRS  Increased levels of inflammatory markers such as IL-6 in critical patients | (7) |
| Deficiency common in elderly | (8) (9) (5) | Disease severe in elderly | (9) |
| Deficiency common in people with co morbids ( diabtes, hypertension) | (9) | Severity in people with co morbids (diabetes, hypertension) | (9) |
| Low levels in people in northern latitude | (3) | High prevelance in people in Northern latitiudes | (3) |
| High levels in Scandinavian diets | (9) (3) | Low prevalence in Scandinavian countries | (9) (3) |
| Reduces viral replication via defensins | (8) |  |  |
| Enhances expression of ACE-2 | (5) | High levels of ACE-2 related to better health outcomes | (5) |
| Deficiency induces a prothrombotic state | (10)  (9) | CAC one of the risk factors for mortality | (9) |
| VDI causes essential hypertension | (9) | Hypertension one of the risk factors for increased severity and mortality | (9) |
| VDI is highly prevalent in dark-skinned persons | (9) | over-representation of African Americans among COVID-19 deaths | (9) |
| Low levels in European countries like Italy, Spain | (3) | Highest mortality rate in Italy | (3) (11) |

1. Lai CC, Shih TP, Ko WC, Tang HJ, Hsueh PR. Severe acute respiratory syndrome coronavirus 2 (SARS-CoV-2) and coronavirus disease-2019 (COVID-19): The epidemic and the challenges. Vol. 55, International Journal of Antimicrobial Agents. Elsevier B.V.; 2020. p. 105924.

2. Vitamin D: A rapid review of the evidence for treatment or prevention in COVID-19 - The Centre for Evidence-Based Medicine [Internet]. [cited 2020 Nov 19]. Available from: https://www.cebm.net/covid-19/vitamin-d-a-rapid-review-of-the-evidence-for-treatment-or-prevention-in-covid-19/

3. Panarese A, Shahini E. Covid‐19, and vitamin D. Aliment Pharmacol …. 2020;

4. Maghbooli Z, Sahraian MA, Ebrahimi M, Pazoki M, Kafan S, Tabriz HM, et al. Vitamin D sufficiency, a serum 25-hydroxyvitamin D at least 30 ng/mL reduced risk for adverse clinical outcomes in patients with COVID-19 infection. PLoS One. 2020 Sep 1;15(9 September).

5. Ilie PC, Stefanescu S, Smith L. The role of vitamin D in the prevention of coronavirus disease 2019 infection and mortality. Aging Clin Exp Res [Internet]. 2020;(0123456789):8–11. Available from: https://doi.org/10.1007/s40520-020-01570-8

6. Jakovac H. COVID-19 and vitamin D-Is there a link and an opportunity for intervention? Am J Physiol - Endocrinol Metab. 2020;318(5):E589.

7. Aranow C. Vitamin D and the immune system. In: Journal of Investigative Medicine. BMJ Publishing Group; 2011. p. 881–6.

8. Grant WB, Lahore H, McDonnell SL, Baggerly CA, French CB, Aliano JL, et al. Evidence that vitamin d supplementation could reduce risk of influenza and covid-19 infections and deaths. Nutrients. 2020;12(4):1–19.

9. Lau FH, Majumder R, Torabi R, Saeg F, Hoffman R, Cirillo JD, et al. Vitamin D Insufficiency is Prevalent in Severe COVID-19. medRxiv. 2020;(504):2020.04.24.20075838.

10. Tian Y, Rong L. Letter: Covid-19, and vitamin D. Authors’ reply. Aliment Pharmacol Ther. 2020;51(10):995–6.

11. Vitamin D affects Covid-19 mortality Pharmaceutical Technology [Internet]. [cited 2020 Nov 19]. Available from: https://www.pharmaceutical-technology.com/comment/vitamin-d-covid-19/
